# A novel circPIK3C2A/miR‐31‐5p/TFRC axis drives ferroptosis and accelerates myocardial injury

**DOI:** 10.1002/mco2.571

**Published:** 2024-06-05

**Authors:** Shuo Miao, Lanting Yang, Tao Xu, Zhantao Liu, Yixiao Zhang, Lin Ding, Wei Ding, Xiang Ao, Jianxun Wang

**Affiliations:** ^1^ School of Basic Medicine Qingdao University Qingdao China; ^2^ Central Laboratory Qingdao Agricultural University Qingdao China; ^3^ Department of Comprehensive Internal Medicine Affiliated Hospital of Qingdao University Qingdao China

**Keywords:** circPIK3C2A, ferroptosis, Iron overload, miR‐31‐5p, TFRC

## Abstract

Iron overload is common in cardiovascular disease, it is also the factor that drives ferroptosis. Noncoding RNAs play an important role in heart disease; however, their regulatory role in iron overload‐mediated ferroptosis remains much unknown. In our study, the iron overload model in mice was constructed through a high‐iron diet, and ammonium iron citrate  treatment was used to mimic iron overload in vitro. We found iron overload induced ferroptosis in cardiomyocytes, which was dependent on the high expression of transferrin receptor (TFRC). MiR‐31‐5p was downregulated during iron overload; it inhibited cardiomyocyte ferroptosis by targeting TFRC. CircPIK3C2A, a highly expressed circRNA in the heart, was upregulated when iron was overloaded. CircPIK3C2A enhanced the expression of TFRC by sponging miR‐31‐5p and promoted ferroptosis during iron overload. Our results reveal a novel mechanistic insight into noncoding RNA‐based ferroptosis and identify the circPIK3C2A/miR‐31‐5p/TFRC axis as a promising therapeutic target for myocardial damage.

## Graphical abstract

CircPIK3C2A/miR‐31‐5p/TFRC axis promotes ferroptosis and exacerbates myocardial injury through inducing iron overload.

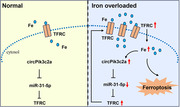

## 1 INTRODUCTION

Cardiovascular disease is a common chronic disease with high morbidity, high mortality, and is difficult to cure. It is a problem that troubles the clinic and needs to be solved urgently. Myocardial injury is an important cause of death from cardiovascular diseases, as it involves the death and loss of cardiomyocytes. For cardiomyocytes, the ability to self‐proliferation is limited, and their loss will lead to rapid decline of heart function and even heart failure. Reducing the loss of cardiomyocytes or inducing their regeneration is one of the strategies to prevent and treat cardiac dysfunction. However, much remains unknown about the mechanism of cardiomyocyte death.

Ferroptosis is an iron‐dependent form of cell death with lipid oxidation as a central feature. The excessive oxidation of lipids is caused by the imbalance of oxidation and antioxidant systems, which does not require specific executive proteins. Its mechanism involves many aspects, including iron metabolism, lipid metabolism, oxidation system, and antioxidant system.^1^ Ferroptosis induced by polyunsaturated fatty acid and the dysfunction of antioxidant system are increasingly revealed.^2^ Iron overload is another key factor that causes ferroptosis, even though it is related to the high expression of transferrin receptor (TFRC), much remains unknown about its upstream regulatory mechanism.^3^, ^4^ In addition, iron deposition in cardiac tissue is a common clinical phenomenon in patients with heart disease, and it is an important factor leading to cardiac dysfunction, such as cardiac insufficiency, ventricular remodeling, and heart failure,^5^, ^6^ It is worth pondering whether ferroptosis is related to cardiac dysfunction caused by iron deposition.

Noncoding RNAs, especially microRNA (miRNA) and circular RNA (circRNA), are involved in the occurrence and development of cardiovascular diseases at both the pretranscriptional and posttranscriptional levels, and are potential targets in clinical diagnosis and therapy. One of the most classical ways in which noncoding RNAs involved in disease development is the sponging action of circular RNA on miRNA, which in turn regulates gene expression.^7^, ^8^ In recent years, several studies report that noncoding RNAs are involved in ferroptosis, especially in cancers. During ferroptosis, the levels of noncoding RNAs are altered and they regulate the process of ferroptosis.^9^, ^10^ Our recent work revealed the role of platelet miR‐223‐3p in cardiomyocyte ferroptosis.^11^ Despite great progress, there are still many gaps in the mechanism by which noncoding RNAs regulate ferroptosis.

To better understand the mechanism of ferroptosis during myocardial injury, the role of noncoding RNAs was analyzed in our study. Our results reveal that iron overload‐mediated ferroptosis in cardiomyocytes is dependent on TFRC and that circPIK3C2A/miR‐31‐5p are upstream regulators of TFRC. Overexpression of miR‐31‐5p alleviates iron overload‐mediated ferroptosis and improves cardiac function.

## 2 RESULTS

### 2.1 Iron overload induces cardiomyocyte ferroptosis and cardiac injury

**FIGURE 1:**
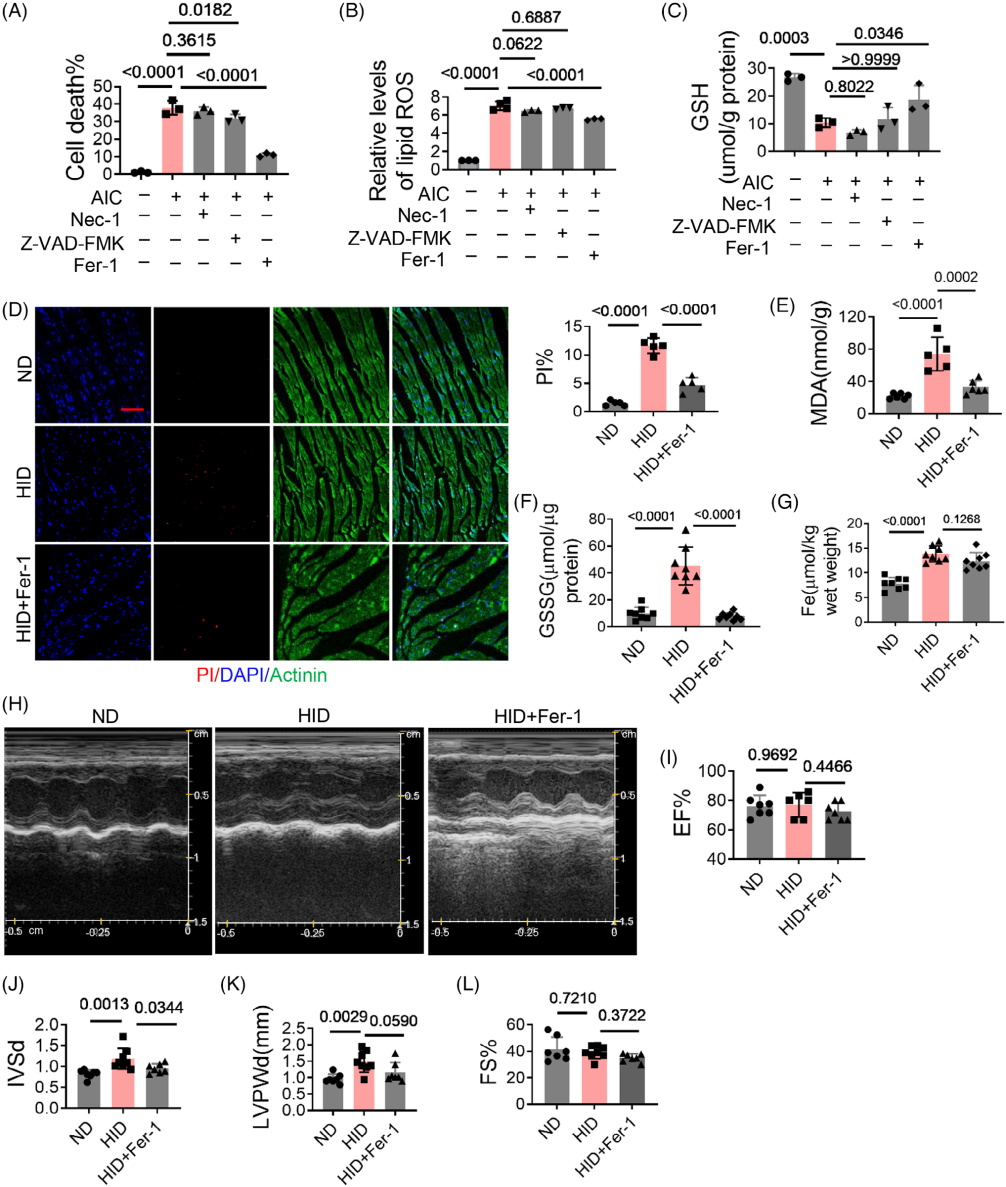
Iron overload induces cardiomyocyte ferroptosis and cardiac injury. (A) The death of H9c2 cells was analyzed by PI staining after AIC treatment for 24 h (*n* = 3). (B) Lipid ROS in H9c2 cells was analyzed by C11 BODIPY 581/591 (*n* = 3). (C) GSH levels in H9c2 cells (*n* = 3). (D) Cell death (*n* = 5); scale bar, 100 µm. (E) MDA levels in hearts (*n* = 7). (F) GSSG levels in heart tissues (*n* = 8). (G) Iron levels in heart tissues (*n* = 8). (H–L) Analysis of cardiac function by echocardiography (*n* = 7). Data are the mean ± SD, and *p* values were determined by using one‐way ANOVA with Bonferroni's multiple comparisons test. Fer‐1, ferrostatin‐1; Nec‐1, necrostatin‐1; AIC, ammonium iron citrate; I/R, ischemia/reperfusion; MDA, malondialdehyde; EF, ejection fraction; FS, fractional shortening; LVPWd, left ventricular posterior wall; IVSd, interventricular septal thickness at diastole.

To mimic iron overload, cardiomyocytes were treated with ammonium iron citrate (AIC). The results showed that with the treatment of AIC, ferroptosis occurred in cardiomyocytes, the percentage of death increased, lipids overoxidized, and GSH level decreased (Figures 1A–C). Ferrostatin‐1(Fer‐1) significantly reduced cell death, improved lipid oxidation, and increased GSH. Necrostatin‐1 (Nec‐1, necrosis inhibitor) did not show significant protective effects. Z‐VAD‐FMK, an inhibitor of apoptosis, also inhibited cell death, but did not affect lipid oxidation and GSH. A mouse model of iron overload was constructed by high‐iron diet (HID) feeding. In HID mice, cell death and GSSG levels in heart tissues as well as malondialdehyde (MDA) in plasma were significantly increased, which were rescued by Fer‐1 (Figures 1D–F). But Fer‐1 had no significant effect on iron levels (Figure 1G). HID mice exhibited structural changes in hearts marked by increased interventricular septal thickness (IVS) and left ventricular posterior wall thickness (LVPW), while Fer‐1 treatment had a remission effect (Figures 1H–L). These results suggest that iron overload induces ferroptosis of cardiomyocytes and cardiac injury.

### 2.2 TFRC participates in iron overload‐induced cardiomyocyte ferroptosis

**FIGURE 2:**
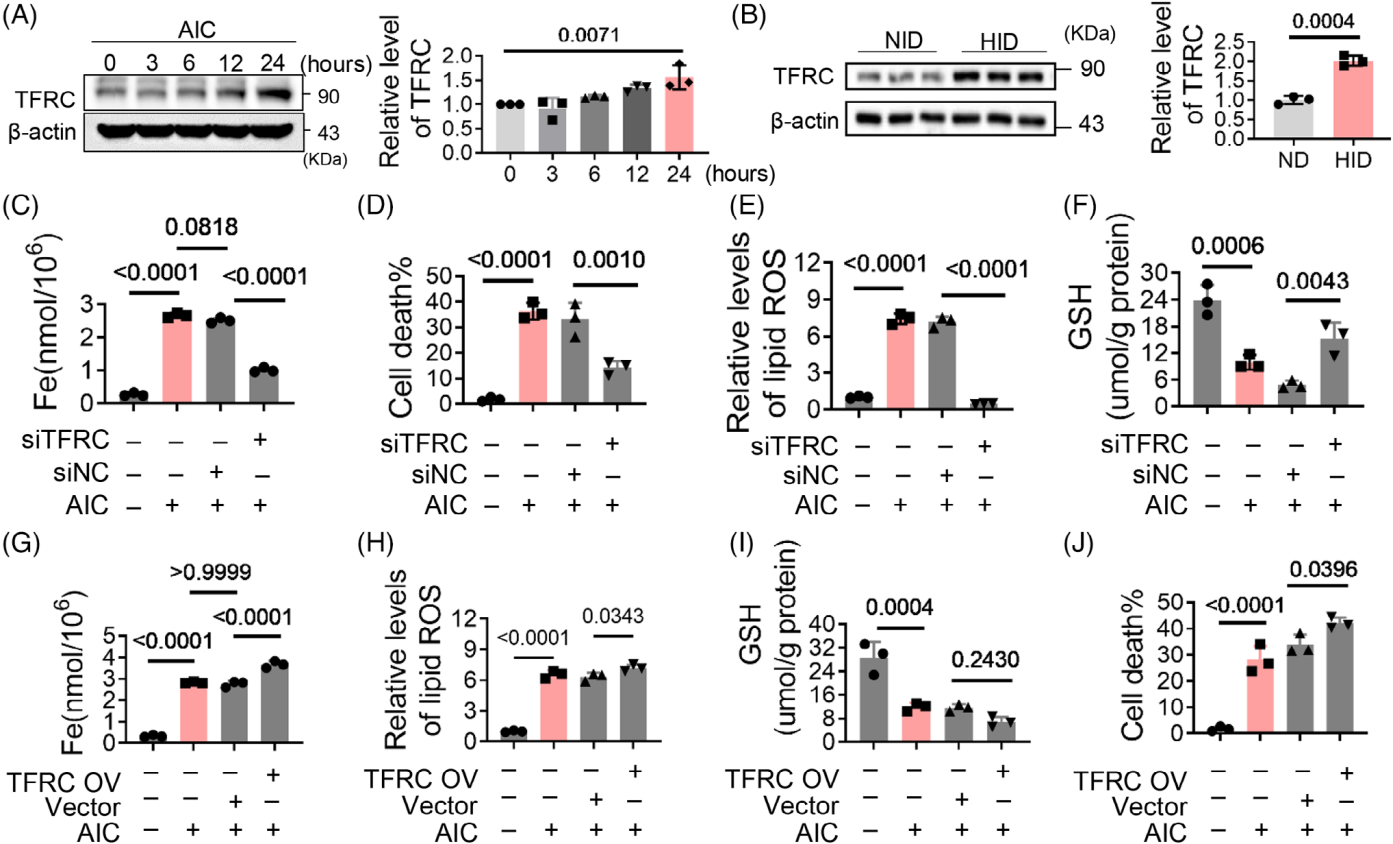
TFRC is involved in iron overload‐induced cardiomyocyte ferroptosis. (A) TFRC protein in H9c2 cells were analysed by western blotting after AIC treatment for indicated hours (*n* = 3). (B) TFRC levels in myocardial tissues (*n* = 3). (C–F) H9c2 cells were cotreated with siTFRC/siNC and AIC for 24 h. (C) Iron levels (*n* = 3). (D) Cell death was analyzed by PI staining (*n* = 3), and the statistical data are shown. (E) Lipid ROS detected by flow cytometry (*n* = 3). (F) GSH levels in H9c2 cells (*n* = 3). (G–J) H9c2 cells were cotreated with TFRC‐overexpressing plasmid/vector and AIC for 24 h. (G) Iron levels in H9c2 cells (*n* = 3). (H) Lipid ROS (*n* = 3). (I) GSH levels in H9c2 cells. (J) Cell death was analyzed by PI staining (*n* = 3), and the statistical data are shown. Data are the mean ± SD, and *p* values were determined by using one‐way ANOVA with Bonferroni's multiple comparisons test. AIC, ammonium iron citrate; ND, normal diet; HID, high‐iron diet.

TFRC, an iron transporter, is involved in iron overload. To assess its contribution to ferroptosis, we first investigated the expression of TFRC. Under the treatment of AIC, its expression gradually increased with the extension of time (Figures 2A and S1A). TFRC levels in HID group were also higher than those in normal diet (ND) group (Figure 2B). This suggests that iron overload upregulates TFRC expression. Further results reveal that TFRC deficiency significantly decreased iron levels in cardiomyocytes (Figures S1B and 2C), meanwhile alleviated the cell death, lipid oxidation and GSH oxidation (Figures 2D–F). The effects of AIC were exacerbated with TFRC overexpression (Figures S1C and 2G–J). These results indicate that TFRC is involved in iron overload‐induced ferroptosis by increasing intracellular iron levels, and iron overload upregulates TFRC expression, which further exacerbates ferroptosis.

### 2.3 MiR‐31‐5p alleviates myocardial ferroptosis by targeting TFRC

**FIGURE 3:**
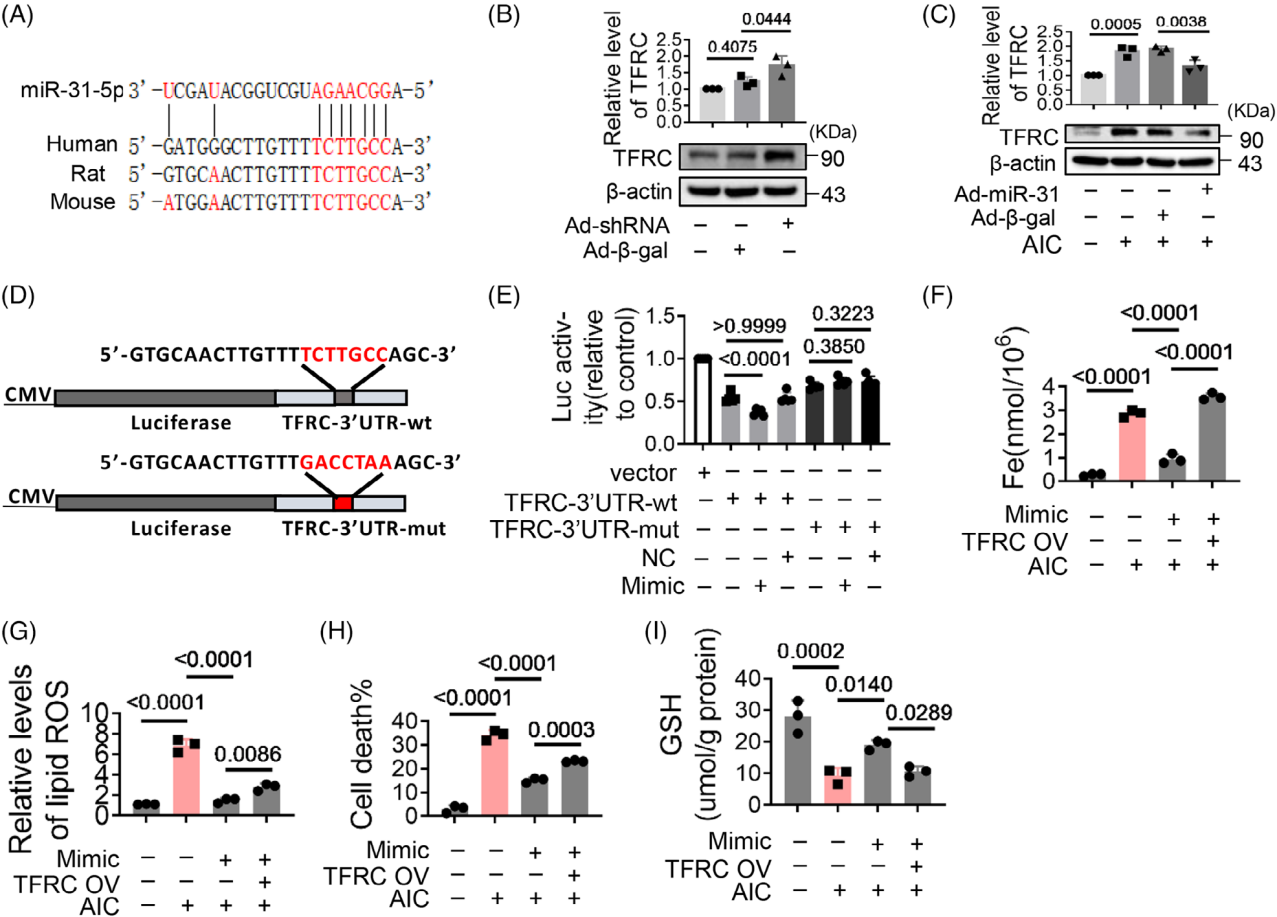
MiR‐31‐5p alleviates AIC‐induced myocardial ferroptosis by targeting TFRC. (A) The binding sites between miR‐31‐5p and TFRC 3′UTR. (B and C) H9c2 cells were treated with adenoviruses for 24 h. The levels of TFRC were analyzed by western blotting. (D) Wild‐type and mutant sequences of TFRC 3′UTR. (E) Luciferase assay. HEK293 cells were cotransfected with miR‐31‐5p mimics/negative control and TFRC‐3′UTR‐wt/TFRC‐3′UTR‐mut, the luciferase activity was analyzed (*n* = 3). (F–I) H9c2 cells were cotransfected with miR‐31‐5p mimic and TFRC‐overexpressing plasmid, and then treated with AIC for 24 h. (F) Iron levels in H9c2 cells (*n* = 3). (G) Lipid ROS (*n* = 3). (H) Percentage of cell death (*n* = 3). (I) GSH levels in H9c2 cells (*n* = 3). Data are the mean ± SD, and *p* values were determined by using one‐way ANOVA with Bonferroni's multiple comparisons test. Ad‐shRNA, miR‐31‐5p shRNA adenoviruses; Ad‐miR‐31, MiR‐31‐5p overexpression adenoviruses; Ad‐β‐gal, adenoviruse vector β‐gal; 3′UTR‐wt, wild‐type of 3′UTR; 3′UTR‐mut, mutant of 3′UTR; mimic, miR‐31‐5p mimics; NC, negative control; AIC, ammonium iron citrate; TFRC OV, TFRC‐overexpressing plasmid.

Next, we analyzed the upstream regulators of TFRC. Bioinformatic analysis suggest that miR‐31‐5p could bind to the 3′UTR of TFRC mRNA (Figure 3A). Experiments show that the expression of TFRC increased significantly when miR‐31‐5p was knocked down (Figures S1D and 3B), while TFRC decreased with miR‐31‐5p overexpression (Figures S1E and 3C). When the 3′UTR of TFRC was mutated, the inhibitory effect of miR‐31‐5p on TFRC was weakened (Figures 3D and E), suggesting miR‐31‐5p inhibits the expression of TFRC by binding to its 3′UTR. Consistent with this, miR‐31‐5p mimics reduced iron deposition, lipid oxidation, cell death, and GSH oxidation. However, these effects were antagonized by TFRC overexpression (Figures 3F–I). These reveal that miR‐31‐5p reduces iron deposition by inhibiting TFRC expression. We found that miR‐31‐5p was downregulated in AIC‐treated cells and HID mice (Figures S2A and B), which may account for the high expression of TFRC during iron overload.

### 2.4 CircPIK3C2A is an upstream regulator of miR‐31‐5p

**FIGURE 4:**
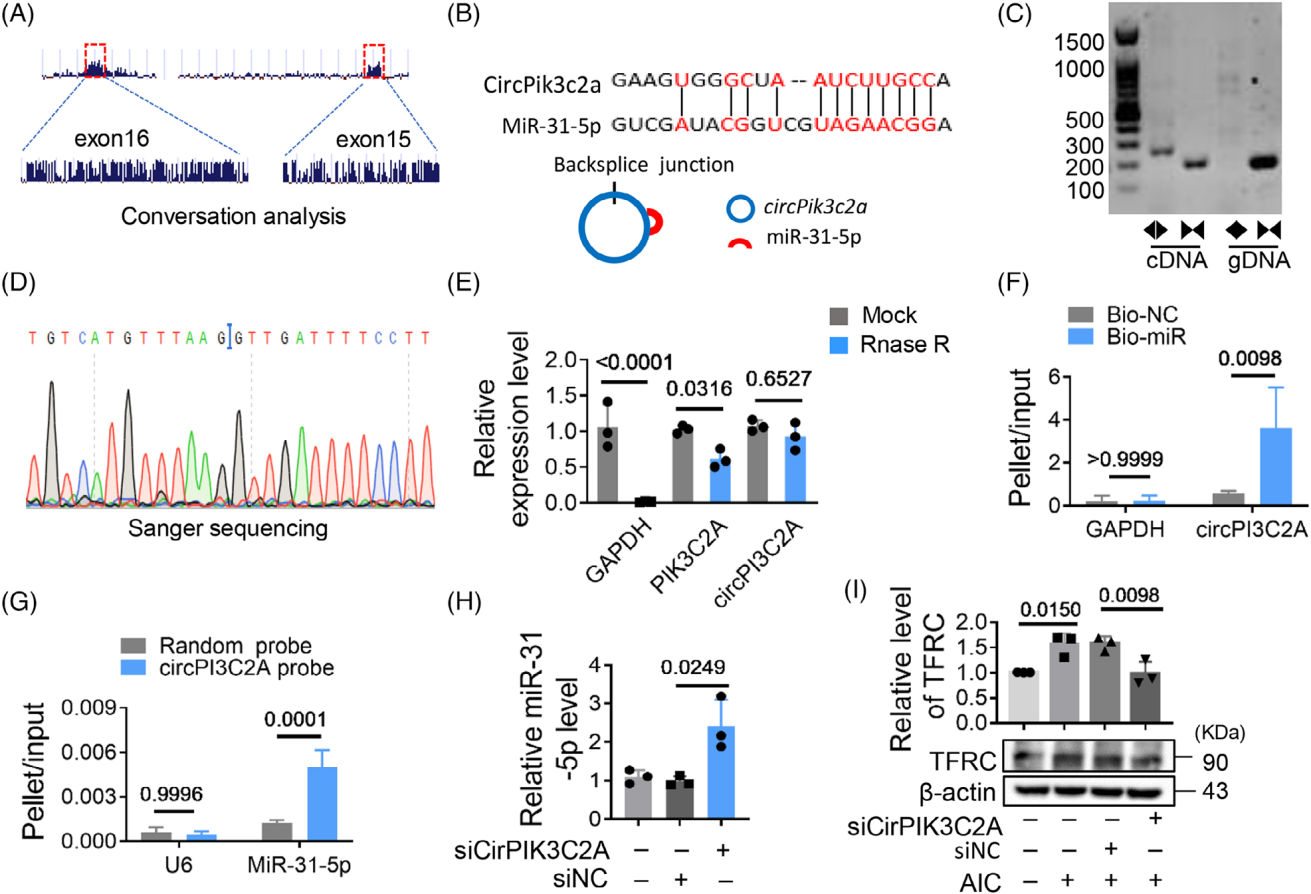
CircPIK3C2A is an upstream regulator of miR‐31‐5p. (A) CircPIK3C2A was generated from exon 15 and 16. (B) The binding sites between circPIK3C2A and miR‐31‐5p. (C) The cyclization of circPIK3C2A was verified by PCR. (D) The head‐to‐tail connection is verified by Sanger‐Seq. (E) RNAs from H9c2 cells were incubated with RNase R or buffer only (Mock). After digestion, the RNAs were purified. The levels of circPIK3C2A and PIK3C2A were analyzed by qRT‐PCR (*n* = 3). (F) Biotin‐labeled miR‐31‐5p mimics or control mimics were transfected into H9c2 cells. The levels of total and streptavidin‐captured circPIK3C2A were analyzed by qRT‐PCR (*n* = 3). The relative projectile/input ratio is calculated. (G) The levels of total and pulled‐down miR‐31‐5p/U6 by the circPIK3C2A/control probe were analyzed by qRT‐PCR (*n* = 3), and the relative pellet/input ratios were calculated. (H) H9c2 cells were transfected with circPIK3C2A siRNA. The level of miR‐31‐5p was analyzed by qRT‐PCR (*n* = 3). (I) H9c2 cells were cotreated with circPIK3C2A siRNA and AIC, and the level of TFRC was analyzed by western blotting (*n* = 3). Data are the mean ± SD, and *p* values were determined by using one‐way ANOVA with Bonferroni's multiple comparisons test. Bio‐miR, biotin‐labeled miR‐31‐5p mimics; Bio‐NC, biotin‐labeled control mimics; siCircPIK3C2A, circPIK3C2A siRNA.

To analyze the reason why miR‐31‐5p was expressed at low levels during iron overload, we screened circRNAs with potential miR‐31‐5p binding sites through an online database (ENCORI). Two potential circRNAs (circPIK3C2A and circSTRN3) were targeted for analysis due to their reported high expression in cardiac tissue.^12^ We found that circPIK3C2A was dramatically elevated in AIC treated cells and HID mice. However, circSTRN3 did not change significantly (Figures S2C and D). The upregulated circPIK3C2A may be associated with the low levels of miR‐31‐5p. CircPIK3C2A is conserved, there is one binding site between circPIK3C2A and miR‐31‐5p (Figures 4A and B). We detected and validated the circularized junction of circPIK3C2A (Figures 4C and D). RNase R degraded linear RNA transcripts (PIK3C2A and GAPDH mRNA), but had no significant effect on circPIK3C2A, further validating its circular nature (Figure 4E). The interaction between circPIK3C2A and miR‐31‐5p was validated by RNA pull‐down assay, the result shows that circPIK3C2A enrichment was significantly higher in miR‐31‐5p group than that in control group (Figure 4F), and the circPIK3C2A probe enriched more miR‐31‐5p than the scrambled probe (Figure 4G). When circPIK3C2A was knocked down, the levels of miR‐31‐5p increased (Figures S2E–G and 4H), but TFRC decreased (Figure 4I). These results indicate that circPIK3C2A binds with miR‐31‐5p and promotes its degradation, which relieves the inhibitory effect of miR‐31‐5p on TFRC.

### 2.5 CircPIK3C2A promotes cardiomyocyte ferroptosis via the miR‐31‐5p/TFRC axis

**FIGURE 5:**
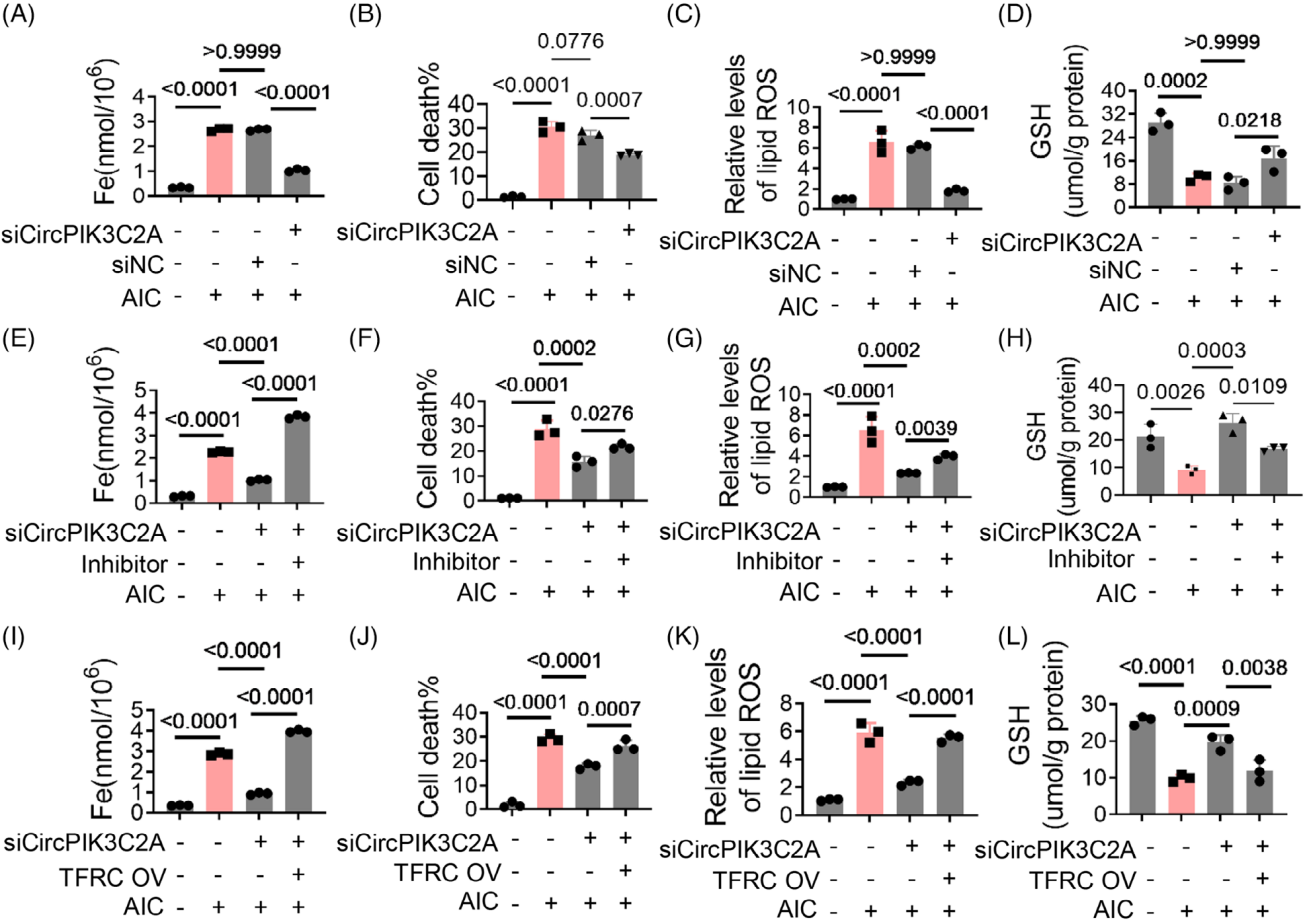
CircPIK3C2A promotes cardiomyocyte ferroptosis via the miR‐31‐5p/TFRC axis. (A–D) H9c2 cells were cotreated with circPIK3C2A siRNA and AIC for 24 h. (E–H) H9c2 cells were cotransfected with circPIK3C2A siRNA and miR‐31‐5p inhibitor and treated with AIC for 24 h. (I–L) H9c2 cells were cotransfected with circPIK3C2A siRNA and TFRC‐overexpressing plasmid, and treated with AIC for 24 h. Iron levels in H9c2 cells (A, E, I, *n* = 3). Percentage of cell death (B, F, J, *n* = 3). Lipid ROS detected by flow cytometry (C, G, K, *n* = 3). GSH levels in H9c2 cells (D, H, L, *n* = 3). Data are the mean ± SD, and *p* values were determined by using one‐way ANOVA with Bonferroni's multiple comparisons test. AIC, ammonium iron citrate; siCircPIK3C2A, circPIK3C2A siRNA; siNC, negative control of siRNA; TFRC OV, TFRC‐overexpressing plasmid.

Next, we analyzed the role of circPIK3C2A in ferroptosis. With the knockdown of circPIK3C2A, iron deposition mediated by AIC decreased significantly, as well as cell death and lipid oxidation, and the decrease of GSH was also alleviated (Figures 5A–D). These results suggest circPIK3C2A is involved in ferroptosis. As expected, miR‐31‐5p inhibitor antagonized the protective effects of siCircPIK3C2A (Figures 5E–H). TFRC overexpression antagonized the effects of siCircPIK3C2A (Figures 5I–L), further revealing miR‐31‐5p/TFRC is the downstream of circPIK3C2A.

### 2.6 MiR‐31‐5p regulates HID‐induced ferroptosis in the heart

**FIGURE 6:**
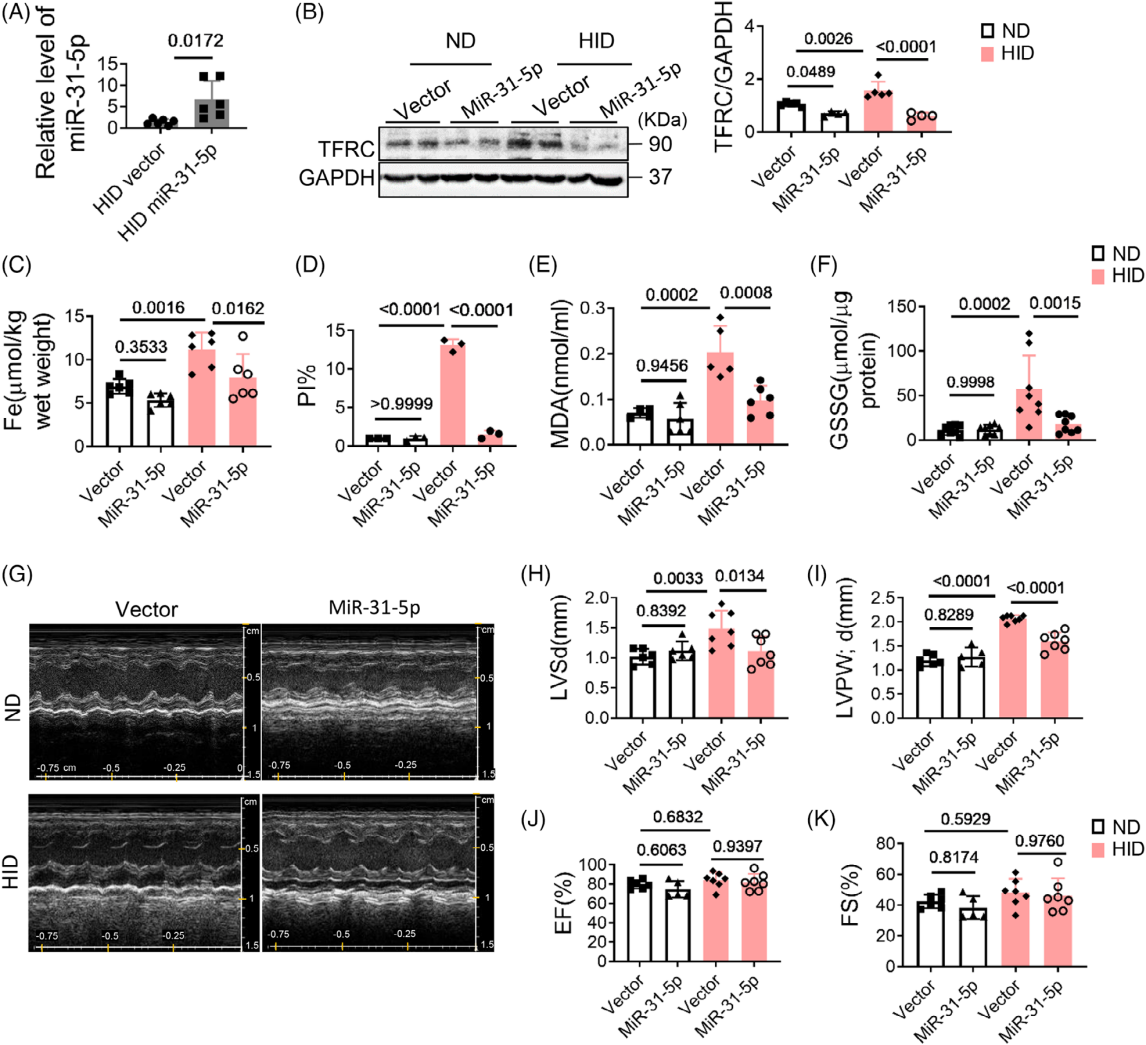
Overexpression of miR‐31‐5p inhibits ferroptosis and improves cardiac function. (A) Adeno‐associated virus overexpressing miR‐31‐5p or vector was injected into mice through the tail vein. The expression efficiency was measured by qRT‐PCR (*n* = 6). (B) The levels of TFRC in mouse hearts were analyzed by western blotting (*n* = 4). (C) Iron levels in mouse hearts (*n* = 6). (D) Percentage of cell death (*n* = 3). (E) MDA levels in plasma (*n* = 6). (F) GSSG levels in heart tissues (*n* = 8). (G–K) Cardiac function was analyzed by echocardiography (*n* = 7). Data are the mean ± SD, and the *p* value was determined by using one‐way ANOVA with Bonferroni's multiple comparisons test. MDA, malondialdehyde; ND, normal diet; HID, high‐iron diet; EF, ejection fraction; FS, fractional shortening; LVPWd, left ventricular posterior wall; IVSd, interventricular septal thickness at diastole.

After confirming that miR‐31‐5p regulates AIC‐induced ferroptosis by targeting TFRC in vitro, we further analyzed its role in vivo. We constructed mice with cardio‐specific high expression of miR‐31‐5p by injecting adeno‐associated virus (pcAAV‐cTnT‐pri‐mmu‐miR‐31‐5p) into the tail vein (Figure 6A). With the high expression of miR‐31‐5p, TFRC levels, iron deposition and cell death in myocardial tissue were significantly reduced (Figures 6B–D and S4A), the levels of MDA and GSSG in myocardial tissues also decreased (Figures 6E and F). In addition, the changes in the hearts were also alleviated to some extent (Figures 6G–K). These data reveal that miR‐31‐5p is a cardiac protectant against ferroptosis, it reduces iron deposition by inhibiting TFRC expression.

### 2.7 MiR‐31‐5p attenuates I/R injury

**FIGURE 7:**
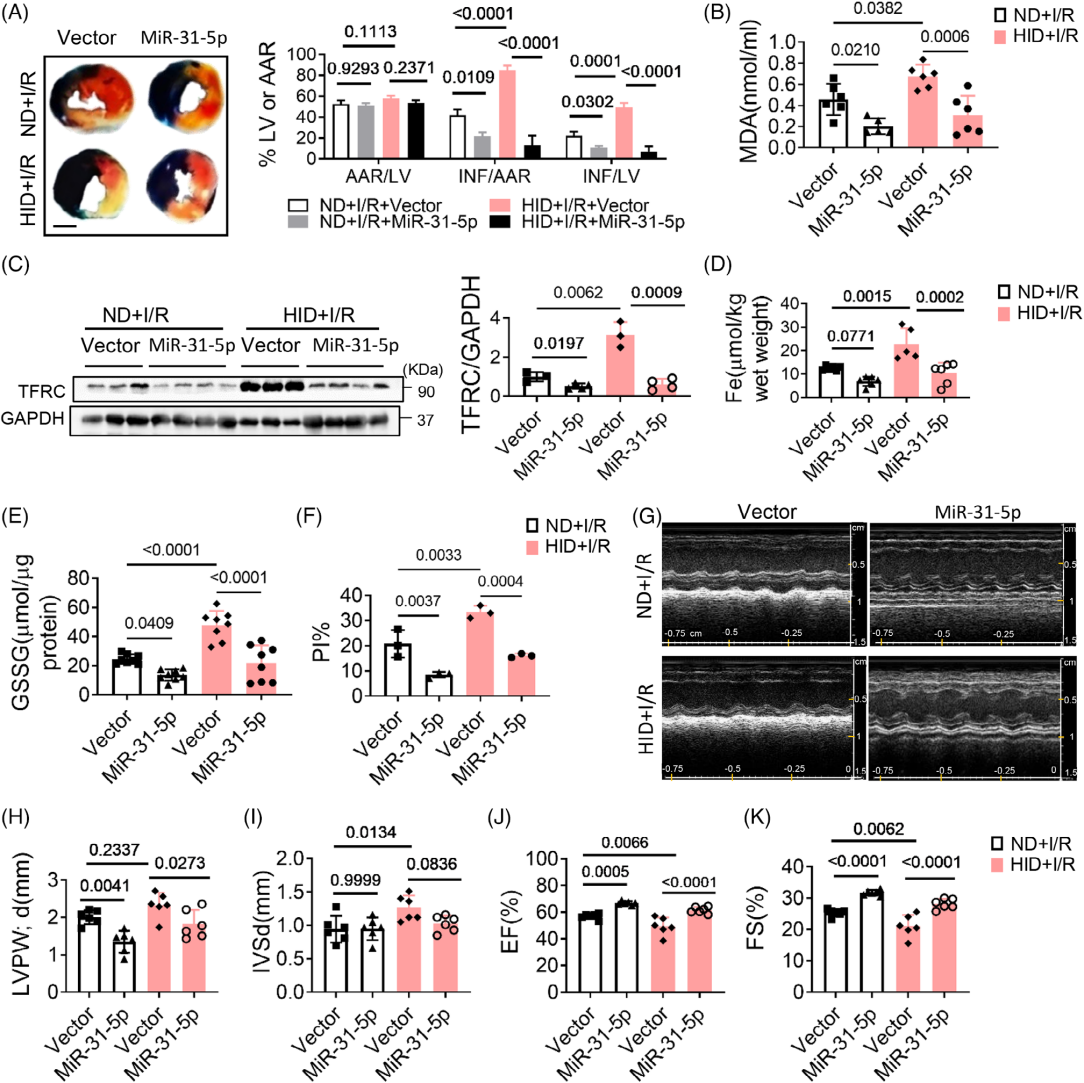
Overexpression of miR‐31‐5p suppresses I/R injury. (A–F) Adeno‐associated virus overexpressing miR‐31‐5p or vector was injected into ND and HID mice before I/R injury. (A) Infarct sizes and representative images are shown (*n* = 3). Scale bars, 2 mm. (B) MDA levels in plasma (*n* = 5). (C) TFRC levels in mouse hearts were analyzed by western blotting (*n* = 3, 4). (D) Iron levels in mouse hearts (*n* = 6). (E) GSSG levels in heart tissues (*n* = 8). (F) Percentage of cell death (*n* = 3). (G–K) Cardiac function (*n* = 6). Data are the mean ± SD, and the *p* value was determined by using one‐way ANOVA with Bonferroni's multiple comparisons test. AAR, area at risk; LV, left ventricular region; INF, infarct area; ND, normal diet; HID, high‐iron diet; I/R, ischemia/reperfusion; MDA, malondialdehyde; EF, ejection fraction; FS, fractional shortening; LVPWd, left ventricular posterior wall; IVSd, interventricular septal thickness at diastole.

Myocardial ischemia–reperfusion (I/R) injury is an unavoidable risk event for acute myocardial infarction. The pathogenic mechanism involves ROS production, iron deposition, calcium overload, and inflammatory reactions. Our experiments confirmed that iron overload induced ferroptosis and myocardial I/R injury, which was mitigated by Fer‐1 (Figures S3A and B). To further reveal the role of miR‐31‐5p in I/R injury, we overexpressed miR‐31‐5p in hearts by injecting adeno‐associated virus prior to constructing the I/R model. The results showed that overexpression of miR‐31‐5p decreased myocardial infarction size (Figure 7A). Moreover, overexpression of miR‐31‐5p decreased MDA, iron content, TFRC, GSSG, and cell death in both ND and HID mice (Figures 7B–F and S4B). Cardiac function was also improved by miR‐31‐5p (Figures 7G–K). Therefore, miR‐31‐5p can also reduce I/R damage by antagonizing ferroptosis.

## 3 DISCUSSION

Our study demonstrated that circPIK3C2A/miR‐31‐5p/TFRC signaling axis promotes ferroptosis and exacerbates myocardial injury by inducing iron deposition. Our results reveal novel mechanistic insights into noncoding RNA‐based ferroptosis and identify circPIK3C2A/miR‐31‐5p are promising therapeutic targets for the treatment of myocardial damage.

Patients who have experienced hemorrhagic shock^13^ or patients with aplastic anemia^14^ often have multiple organ dysfunction due to ischemia and hypoxia, timely and effective blood transfusion is the key for treatment. However, these patients suffered myocardial damage to varying degrees after treatment due to iron deposition.^15^ Consistent with our research, several studies also showed that a HID resulted in severe cardiac injury and hypertrophic cardiomyopathy with molecular features typical of ferroptosis,^16^ and excess iron could induce cardiac ferroptotic cell death as efficiently as erastin (50 µM) and RSL3 (1 µg/mL).^17^ Another study showed that iron overload via heme degradation in the endoplasmic reticulum triggered ferroptosis in myocardial I/R injury.^18^ It has been reported that iron overload‐induced autophagy led to ferroptosis through degradation of ferritin and induction of TFRC expression.^19^ Consistent with others, we also found that iron overload promoted elevated TFRC levels. High expression of TFRC intensifies iron uptake, which may lead to a vicious cycle of iron overload. Iron overload may also be a secondary effect initiated with the onset of ferroptosis. In doxorubicin (DOX)‐induced cardiac injury, deposition of DOX–Fe^2+^ complex in mitochondria leads to mitochondria‐dependent ferroptosis,^20^ DOX also induced high expression of TFRC.^21^, ^22^ In addition, TFRC was reported to induce macrophage activation and infiltration through STAT3‐Ccl2 signaling pathway, promoting the development of heart failure.^23^ In our study, iron levels were significantly increased in I/R group (6.926 ± 0.3414 vs. 12.52 ± 0.4135; Figures 6C and 7D), the combination of HID and I/R resulted in significant myocardial damage and cardiac dysfunction. Beyond the high expression of TFRC, it remains to be further investigated whether there are other mechanisms that mediate iron overload.

MiR‐31‐5p is a highly conserved noncoding RNA involved in a variety of diseases, including sepsis^24^ and colorectal cancer.^25^ And it exhibits different regulatory effects in heart diseases. It promotes arrhythmia by targeting neuronal  nitric oxide synthase^26^ and participates in myocardial hypertrophy by targeting PRKCE.^27^ Unlike previous studies, our current study reveals that miR‐31‐5p alleviated I/R damage and antagonized iron deposition by targeting TFRC. In mitochondria‐dependent ferroptosis, deposition of DOX–Fe^2+^ complex in mitochondria induced excessive lipid peroxidation,^20^ the specific mitochondrion‐targeted antioxidant Mito‐TEMPO, but not TEMPO (a nonspecific antioxidant), attenuated ferroptosis.^28^ Additional studies are needed to determine whether mitochondria‐dependent ferroptosis is affected by miR‐31‐5p.

As sponge RNAs, circRNAs interact with miRNAs, regulating their levels and influencing the expression of downstream target genes. We found that high levels of iron induced circPik3c2a expression but reduced miR‐31‐5p levels. Knockdown of circPik3c2a significantly increased miR‐31‐5p levels. In some cases, targeting binding leads to miRNA decay.^29^ We hypothesize that circPik3c2a acts as an abundant and highly complementary target RNA, which triggers miR‐31‐5p degradation. In other cases, circRNAs act as sponges for miRNAs without affecting their levels.^30^ However, the molecular mechanisms by which miR‐31‐5p is downregulated still need further study.

Recently, several studies have shown a strong correlation between ferroptosis and other types of cell death. For example, quercetin‐induced tumor cell death includes ferroptosis and apoptosis, with ferroptosis preceding apoptosis. Ferroptosis‐mediated BID cleavage promoting apoptosis.^31^ Erastin, an inducer of ferroptosis, also caused BID transactivation to mitochondria and enhanced mitochondrial fragmentation in neuronal cells.^32^ However, these hallmarks of mitochondrial demise are also mentioned in other modes of cell death, such as oxytosis, a paradigm of cell death induced by Xc^−^ inhibition.^32^ In our study, apoptosis inhibitors also inhibited iron overload‐induced cell death, although it was weaker than ferroptosis inhibitor. Iron overload also induces apoptosis in cardiomyocytes,^33^, ^34^ and the mechanism involves mitochondrial dysfunction and mitochondrial biogenesis alteration. Whether there is a relationship between apoptosis and ferroptosis in cardiomyocytes during iron overload remains to be studied further.

## 4 MATERIALS AND METHODS

### 4.1 Cell experiment

The H9c2 cell line (American Type Culture Collection) was cultured in DMEM (HyClone, USA) supplemented with 10% fetal bovine serum at 37°C in a humidified atmosphere containing 5% CO_2_ and 21% O_2_. Cells were treated with 500 µM AIC for 24 h except as otherwise indicated. Transient transfections were conducted with siTFRC (75 pmol), TFRC plasmid (1.5 µg), shCircPIK3C2A (75 pmol), miR‐31‐5p mimic (75 pmol), or antagomiR‐31‐5p (75 pmol). After 24 h, cells were collected for analysis. All sequence information is detailed in Supporting Information.

### 4.2 Animal experiments

C57BL/6 mice (4 weeks, male) were purchased from HFK BIOSCIENCE Co., LTD. (Beijing, China) and housed in SPF environment. Unless stated otherwise, the mice were fed a ND containing 158.60 mg iron/kg (KEAOXIELI FEED Co., LTD. Beijing, China). The HID contains 8.3 g carbonyl iron/kg. The mice were fed HID or ND for 8 weeks. For Fer‐1 treatment, mice were injected intravenously with Fer‐1 (2.5 µmol/kg body weight) every other day during the last week and then sacrificed. To overexpress miR‐31‐5p, mice were injected intravenously with pcAAV‐cTnT‐pri‐mmu‐miR‐31‐5p (miR‐31‐5p, 1.8 × 1012 v.g./mL, 160 µL/mouse) last week and then sacrificed. For the I/R experiment, the HID and ND mice underwent I/R surgery at the end of week 6. The mice were subjected to 30 min of LAD ligation followed by 3 h of reperfusion. Cardiac function was assessed 2 weeks after surgery. Mice were anesthetized by intraperitoneal injection of 1% pentobarbital sodium and euthanized by bleeding. All studies were approved by the Committee of the Ethics of Animal Experiments of Qingdao University (QDU‐AEC‐2022500).

### 4.3 Immunofluorescence

The animals received PI injections of 10 mg/kg to label necrotic cells after the termination of I/R surgery. The myocardial tissues were serially sectioned and then analyzed as previously described.^11^

### 4.4 MiRNA target analysis

TargetScan was used to analyze the potential regulators of TFRC. Enter species (human), Gene (TFRC) in the user interface and select the representative (most prevalent) transcript of TFRC (ENSG00000072274.8). Potential conserved sites for MiR‐31‐5p will be found (position 2564−2571 of TFRC 3′ UTR, 8mer, context++ score −0.48, context++ score percentile 99 and predicted relative *K*_D_ −4.633).

### 4.5 Quantitative real‐time PCR

The total RNA was extracted with Trizol reagent and reverse transcribed with reverse transcriptase (Takara, Tokyo, Japan). Stem‐loop quantitative real‐time PCR (qRT‐PCR) for miR‐31‐5p was performed as previously described,^11^ and its expression was normalized to U6. Other genes were normalized to GAPDH. See Supporting Information for primer information.

### 4.6 Analysis of cell death and lipid ROS

Cells were treated with 500 µM AIC, 2 µM Fer‐1 (MCE; HY‐100579), 10 µg/mL Nec‐1, or 10 µg/mL Z‐VAD‐FMK for 24 h. PI and DAPI staining were used for cell death analysis. Fluorescence microscopy was used to analyze PI‐positive cells. The analysis of lipid ROS was performed as previously described,^11^ in brief, 5 µM C11BODIPY 581/591 (MCE; HY‐D1301) incubated the cells at 37°C for 1−2 h. The cells were collected and then analyzed by flow cytometry (BD; ACCURI C6).

### 4.7 Pull‐down assay

Pull‐down assay with biotinylated miRNA was performed as previously described.^35^ In brief, miR‐31‐5p and scrambled single‐strand RNAs were labeled with 5′‐biotin. H9c2 cells were transfected with biotinylated miRNAs. The cells were lysed in the lysate buffer. And 50 µL of the samples was aliquoted for input. The remaining lysates were incubated with streptavidin agarose beads (Invitrogen). The beads were washed with ice‐cold lysis buffer, low‐salt buffer, and high‐salt buffer. The bound RNAs were purified with Trizol for analysis.

Pull‐down assay with circPIK3C2A probe was performed as previously described.^30^ In brief, the biotinylated DNA probe was synthesized and dissolved in binding buffer. The probes were incubated with streptavidin‐coated agarose beads to generate probe‐coated beads. Lysates of H9c2 cells were incubated with probe‐coated beads, and RNA complexes bound to the beads were eluted and extracted. The detection sequence is detailed in the Supporting Information.

### 4.8 Western blot

Protein was separated using 10% SDS‐PAGE gels. Run electrophoresis at 70 V for 1 h and 100 V for 2 h. The protein was transferred to the NC membrane at 300 mA for 70 min. Primary antibodies include TFRC (ABclonal; catalog A5865), GAPDH (Abcam; ab125247), and β‐actin (Sigma–Aldrich; T8203, clone AA13). Pictures was quantified using Image J software.

### 4.9 Analysis of myocardial infarct size

To analyze the infarct size, 1% Evans blue dye (Sigma) was injected into jugular vein of the mice, the heart was taken and cut transverse continuously into 2 mm thick slices. Then, the slices were incubated in 2% TTC (Sigma) at 37°C for 10−30 min under dark conditions. After washing in PBS, they were fixed by 4% paraformaldehyde (Servicebio; G1101). Images were captured and analyzed by image J (NIH, Boston, USA).

### 4.10 Analysis of iron levels

The iron levels in samples were analyzed by Total iron Colorimetric Assay Kit (Elabscience; E‐BC‐K772‐M). The samples (200 µL/well) were added to the plate, and then 100 µL of chromogenic reagent was added. The plate was incubated at 37°C for 40 min. OD values were determined at 593 nm.

### 4.11 Echocardiography

Cardiac function was evaluated using a small animal echocardiograph (VINNO; VINNO6 LAB) equipped with a 23‐MHZ X10‐23L scanning head. The structure and function of hearts were analyzed by a series of parameters collected, including heart rate, diastolic interventricular septum (IVSd), % fractional shortening (%FS), diastolic LVPW (LVPWd), and ejection fraction (EF).

### 4.12 Statistical analysis

Statistical analysis was conducted by GraphPad Prism 9. Shapiro‐Wilk normality test was performed before analysis. Student's *t*‐test was used to compare two groups. One‐way ANOVA was performed for three or more groups. Kruskal–Wallis test was used for nonnormally distributed data. Data are shown as the mean ± SD, *p *< 0.05 was considered statistically significant.

## AUTHOR CONTRIBUTIONS

J. X. W., W. D., and X. A. designed the study. S. M., L. T. Y., and T. X. performed the experiments. Z. T. L., Y. X. Z., and L. D. developed the methodology. W. D. and X. A. performed the statistical analysis. All the authors have read and approved the final paper.

## CONFLICT OF INTEREST STATEMENT

All authors have no conflict of interest to declare.

## FUNDING INFORMATION

This work was supported by the Natural Science Foundation (Project No. 82270301, 82000290, and 82100285).

## ETHICS STATEMENT AND CONSENT TO PARTICIPATE

All animal studies were approved by the Committee of the Ethics of Animal Experiments of Qingdao University (QDU‐AEC‐2022500).

## Supporting information

Supporting Information

## Data Availability

The data that support the findings of this study are available from the corresponding author upon reasonable request.
