# Supplementary material for: A novel circPIK3C2A/miR‐31‐5p/TFRC axis drives ferroptosis and accelerates myocardial injury
Source: MedComm (2020). 2024 Jun 5;5(6):e571. [Article in Catalan] doi: 10.1002/mco2.571 (PMC11151151; doi:10.1002/mco2.571)
Supplement: Supplementary file 1 — Supporting Information [file MCO2-5-e571-s001.pdf]

# Supporting information

## A novel circPIK3C2A/miR-31-5p/TFRC axis drives ferroptosis and accelerates myocardial injury

Shuo Miao[1], Lanting Yang[1], Tao Xu[2], Zhantao Liu[1], Yixiao Zhang[1], Lin Ding[1], Wei Ding[3]*, Xiang Ao[1]*, Jianxun Wang[1]*

[1] School of Basic Medicine, Qingdao University, Qingdao, China.

[2] Central Laboratory, Qingdao Agricultural University, Qingdao, China

[3] Affiliated Hospital of Qingdao University, Qingdao, China.

* Correspondence: Jianxun Wang (wangjx@qdu.edu.cn), Xiang Ao (xiangao2016@163.com) or Wei Ding (dingwei@qdu.edu.cn).

## I. Sequence information of primers and siRNAs

***Primers for qPCR***

Rat miR-31-5p RT primer:

GTCGTATCCAGTGCAGGGTCCGAGGTATTCGCACTGGATACG ACCAGCTA

Rat miR-31-5p primers: forward, 5'-CGAGGCAAGATGCTGGCA-3'

reverse, 5'-AGTGCAGGGTCCGAGGTATT-3',

Rat Pik3c2a primers: forward, 5'- AGATGATGAAGCACCTGTGGATT-3',

reverse, 5'-CGGTGCTGGTTTTCAGTTTGT -3'.

Rat circPik3c2a divergent primers forward, 5'- AATGCCGGGTTCCTCTCAAACCA -3',

reverse, 5'- TCCAGTGCTTCCAGCTTGTATTGC -3'.

Rat circPik3c2a convergent primers forward, 5'- GATAAGGCCTTTTTGTGGGAAAA -3',

reverse, 5'- CATCAAGGAGCTCCAATGCA -3'

Rat circPik3c2a primers forward, 5'- AATGCCGGGTTCCTCTCAAACCA-3',

reverse,5'- TCCAGTGCTTCCAGCTTGTATTGC -3'.

Rat TRFC primers forward,5'-AGTAGGAGCCCAGAGAGACGCTTGG-3',

reverse,5'- CACTCAGTGGCACCAACAGCTCCAT-3',

U6 primers forward, 5'-CTCGCTTCGGCAGCACA-3',

reverse, 5'-AACGCTTCACGAATTTGCGT-3'.

Rat GAPDH primers forward, 5'-GTCGTGGAGTCTACTGGCGTCTTCA3',

reverse, 5'TCGTGGTTCACACCCATCACAAACA-3'.

Mouse GAPDH primers forward, 5'- AGGTCGGTGTGAACGGATTTG-3',

reverse, 5'- TGTAGACCATGTAGTTGAGGTCA-3'.

***Sequence information of siRNAs***

Rat siTFRC sense: 5'- CCUAAAUCUUCUCGCUUAUTT-3',

anti-sense: 5'- AUAAGCGAGAAGAUUUAGGTT-3'.

Rat sh-circPik3c2a-1: sense, 5'-CAUGUUUAAGGUUGAUUUUTT-3'

anti-sense, 5'-AAAAUCAACCUUAAACAUGTT-3'

Rat sh-circPik3c2a-2 sense, 5'-UGUUUAAGGUUGAUUUUCCTT-3'

anti-sense, 5'-GGAAAAUCAACCUUAAACATT-3'

***Sequence information of mimic and antagomiR***

Rat miR-31-5p mimic: sense: 5'-AGGCAAGAUGCUGGCAUAGCUG-3',

anti-sense: GCUAUGCCAGCAUCUUGCCUUU

Rat miR-31-5p-antagomiR: CAGCUAUGCCAGCAUCUUGCCU

***Sequence information of probes***

The scrambled control probe was: 5'-biotin-TCATGTTTAAGGTTGATTTTCC-3'.

The sequence of rat circPik3c2a probe was: 5'-biotin-GGAAAATCAACCTTAAACATGA-3'.

The sequence of rat miR-31-5p probe: 5'-Biotin-AGGCAAGAUGCUGGCAUAGCU-3'.

## II. Supplementary Figures and Legends

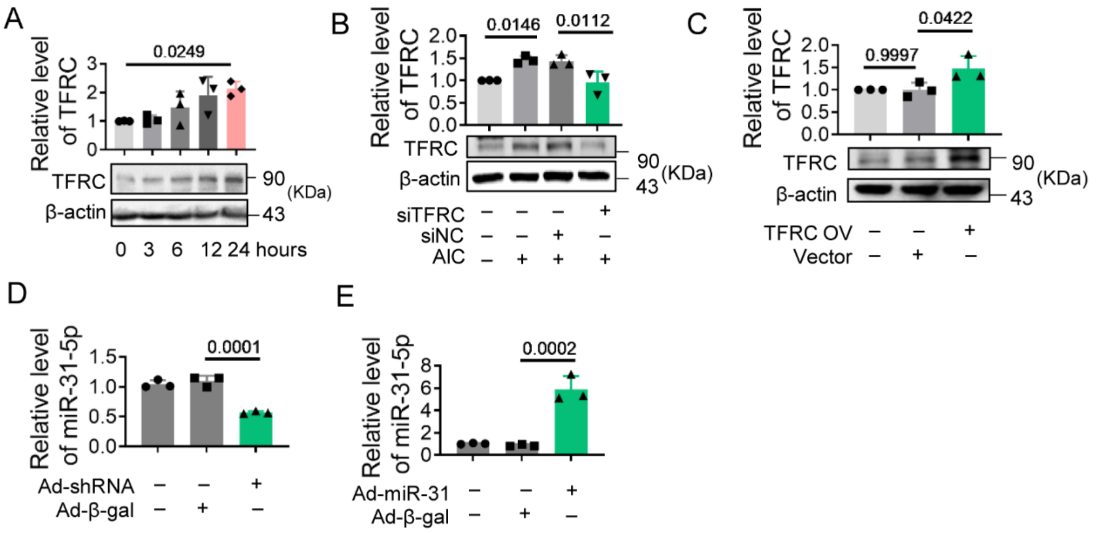


**Figure S1. Knockdown and overexpression efficiency of TFRC and miR-31-5p.** (A) The expression of TFRC in primary cardiomyocytes was analyzed by western blotting (n=3). (B and C) The expression of TFRC in H9c2 was analyzed by western blotting (n=3). TFRC was cloned into the pcDNA3.1 vector. Transfection was performed for 24 hours using Lipofectamine 3000. (D and E) The levels of miR-31-5p were analyzed by qRT-PCR (n=3). MiR-31-5p shRNA adenoviruses and overexpressing adenoviruses infected H9C2 cells for 24 hours. Data are mean ± SDs, *p* values were determined by using 1-way ANOVA with Bonferroni multiple comparisons test. siTFRC: TFRC shRNA; siNC: negative control. TFRC OV: TFRC overexpression plasmid; Ad-shRNA: miR-31-5p shRNA adenoviruses; Ad-miR-31: miR-31-5p overexpressing adenoviruses; Ad-β-gal: vector adenoviruses.

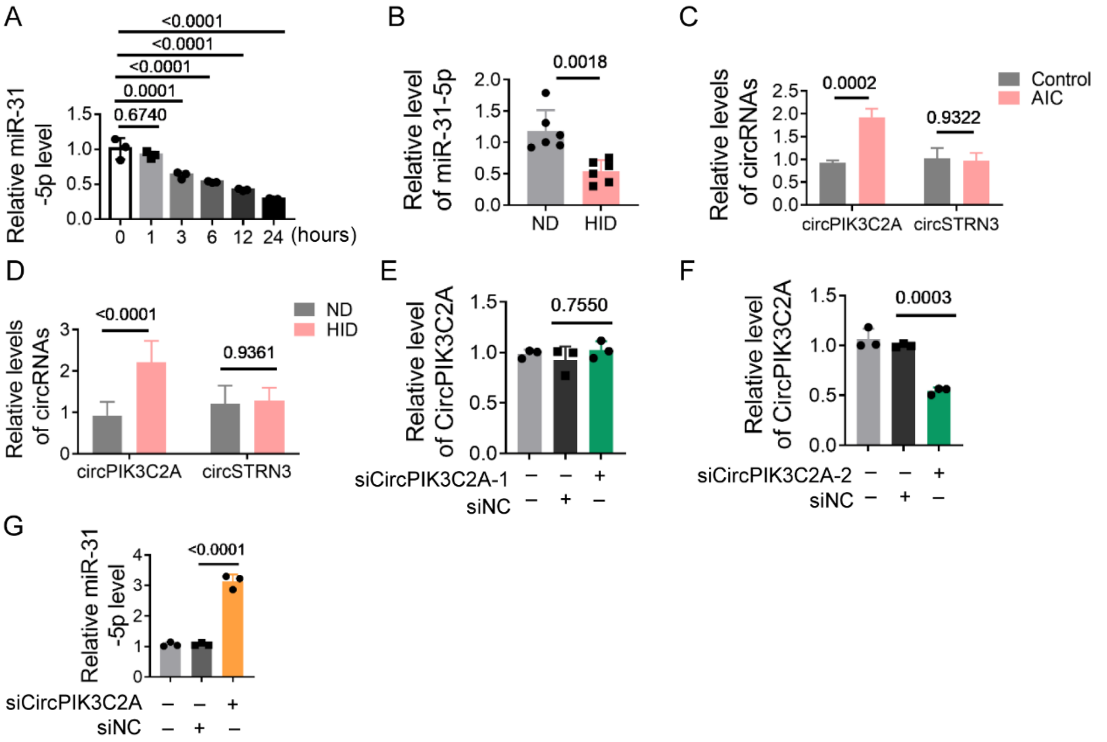


**Figure S2. Analysis of miR-31-5p and circPIK3C2A levels.** (A) H9c2 cells were treated with AIC

for indicated hours and the levels of miR-31-5p was analyzed by qRT-PCR (n=3). (B) The levels of miR-31-5p in ND and HID feeding mice (n=6). (C and D) The levels of circPIK3C2A and circSTRN3 in AIC treated H9c2 cells (n=3) and HID feeding mice (n=6). (E and F) The knockdown efficiency of circPIK3C2A siRNAs (n=3). (G) The level of miR-31-5p in primary cardiomyocytes (n=3). Data are mean ± SDs, *p* values were determined by using 1-way ANOVA with Bonferroni multiple comparisons test. ND: normal diet; HID: high-iron diet; AIC: ammonium iron citrate.

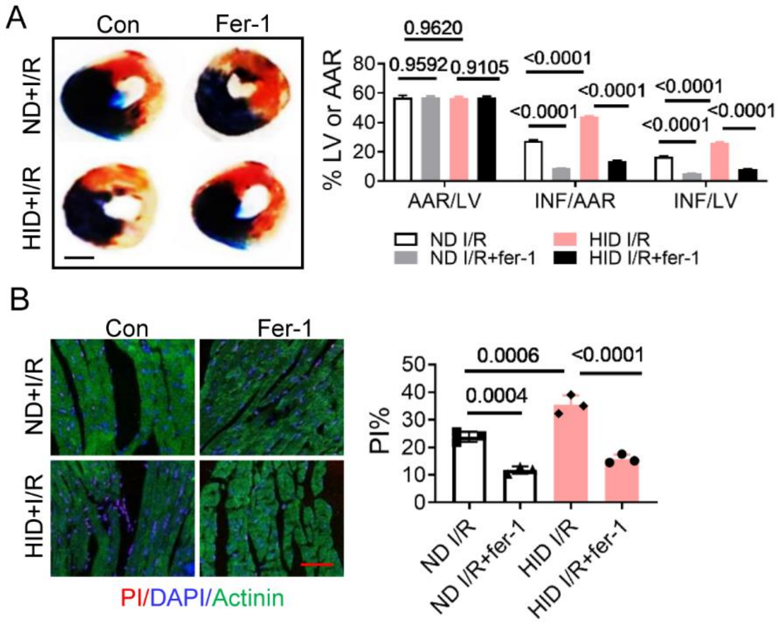


**Figure S3. Iron overload exacerbates myocardial ischemia-reperfusion injury.** The ND and HID mice were injected with Fer-1 through the tail vein four times, once every other day, followed by I/R surgery. (A) Infarct sizes and representative images of midventricular myocardial slices were shown (n = 3). Scale bars, 2 mm. (B) Percentage of cell death was analyzed by PI (n=3), scale bar, 100 μm. Data are mean ± SDs, *p* values were determined by using 1-way ANOVA with Bonferroni multiple comparisons test. ND: normal diet; HID: high-iron diet; I/R: Ischemia/Reperfution. AAR: area at risk; LV: left ventricular region; INF: infarct area.

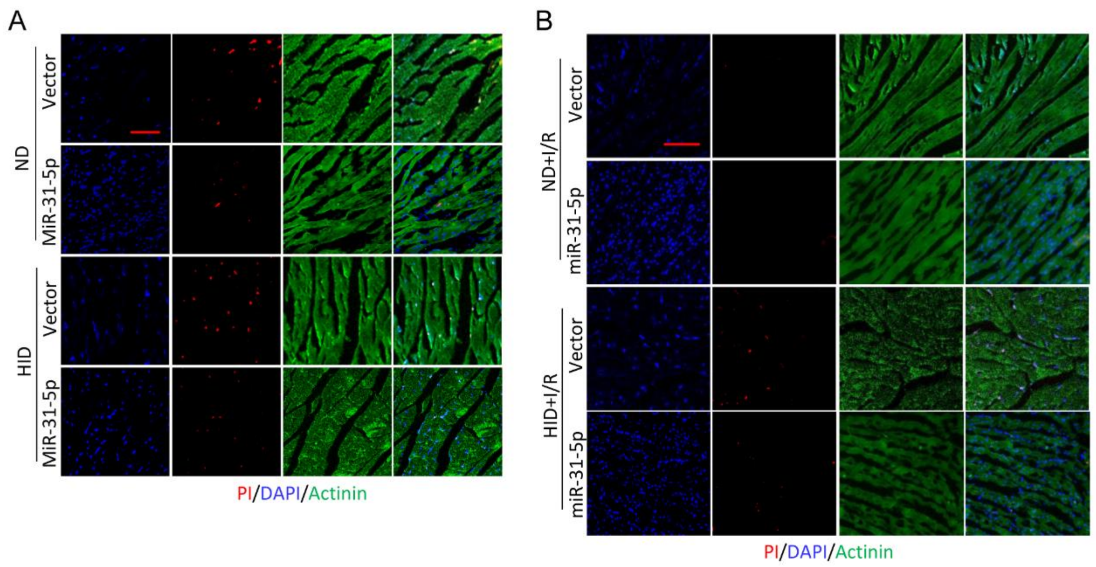


**Figure S4. Cell death in heart tissues was analyzed by PI.** The ND and HID mice were injected with AAV-miR-31-5p or vector through the tail vein (or followed by I/R surgery). Cardiomyocytes were labelled with α-actinin (green) and the representative images are shown. N=3, scale bar, 100 μm.
